# Supplementary material for: Compliance with COVID-19 preventive measures among chronic disease patients in Wolaita and Dawuro zones, Southern Ethiopia: A proportional odds model
Source: PLoS One. 2022 Oct 26;17(10):e0276553. doi: 10.1371/journal.pone.0276553 (PMC9604994; doi:10.1371/journal.pone.0276553)
Supplement: Attachment — Submitted filename: Compliance with COVID-19 preventive measures_august22.pdf [file pone.0276553.s005.pdf]

I like the article. Those are some suggestions. The thing that bothers me is the n number. Size sampled calculate to be 423, the get 419, and only work with 417. I don´t see clearly how you get to 417.

Line 57: "sever form" , probably should be "sever**e**"
Line 58: Space after the dot "(10).Studies"

Line 60 "while a chronic respiratory disease was also indicated in the list". **This sentence is a bit alone and could be fused with the previous one** e.g. "most frequent [**or** some of the most frequent] chronic conditions were hypertension, diabetes, and chronic respiratory diseases … "

Line 67 It was also proven effective → It also has been proven effective

Line 80 (and others along the text): **the apostrophes denoting possession are straight** "experts`..." should be experts'...". Also used that way in other parts of the text. 401 participants` 429

Line 81: **"community" is singular**, therefore "community was" (not were)

Line 94 (and other parts of the text) : i**n the text the use of symbols like < , > ,** = should be avoided, but can be used in tables. 144, 278

Line 111: "so as the finding can help to highlight WASH potential gaps in the study area." → **maybe it would be better to shorten it to better understand it to** "~~so as the finding can help~~ to highlight WASH potential gaps in the study area."
Also, I don't understand why WASH is in capital letters.

144
"who were older than 18 years" or "18 years or older"?
they had chronic diseases → who had chronic diseases → or even "who had hypertension, diabet…. and.."
146 Whereas unable to communicate → Whereas patients unable..

148 Sample size. It surprised me that after calculating a **required sample of 423**, you got 419 and later ended with 417 . It was a good idea to add a 10% non response rate. Have you considered adding a patient flow chart ?

189 … by adding each score out of 44 → by adding each score up to 44??

197 Ye/No → Ye**s**/no?

206 … a higher score (2 point) → the highest score??

214*215 This questionnaire was attested for content validity by health education and public health experts. →**bibliography** supporting that quote?

Results
271 **About the n**, how come you calculated a study size of 423 and only got 419 patients. What happened with the other 4?

273 majorities of them → majority of them
274 …, and employed 282 → and were employed . Here employed is not used as verb but as an adjective.

274-276 In this study, television was mentioned as a major 362 (86.8%), while the website was mentioned as the least 96 (23.0%) source of information about COVID-19 preventive measures.
Changing the order of one line improves its understanding → In this study, television was mentioned as a 275 major 362 (86.8%)source of 276 information about COVID-19 preventive measures , while the website was mentioned as the least 96 (23.0%) .

279 It surprised me to see all the tables with the year 2021 on it. Not used to see that.
Table 1 When you say "Age in years" → "Age (years)"
In the table it is appropriate to use symbols >, < and so on

295 TAble 1 and table 2: n=419 but when you get to line 295 you say "417 had information about covid-19 preventive measurements". How do you get to this n? Much later in the line 352 you say "almost all (417 out of 419) participants had information about covid.

297 More than a half; (59.0%) of participants → More than half of the participants (59,0%)
The semicolon (;) should be erased

298 (51,6%) → erase parenthesis

346 In this study, the determined compliance level and the identified predictors will have a valuable contribution to the current level of knowledge.
I believe this sentence should be rewritten for better understanding and also fused with the next one →

The determination of the level of compliance with the covid19 preventive measures and its predictors in patients with chronic illnesses are a valuable contribution that will help….

352 417/419 → 417 out of 419

354 majorities of the particip →the majority of the participants

360 ….

364 Out of the blue → used more frequently in an informal way → might be better to use “Unexpectedly” or similar. Your pick

365 - 366 responded, blood transfusion as a means → responded blood transfusion was a …
responde that sexual intercourse as another → was another..

366 Findings from Ethiopia → Other studies from Ethiopia ….

367 Findings from Ethiopia and elsewhere showed that (12.38% and 7.7%) of participants responded to blood transfusion, while (9% and 7.4%) of participants, responded to sexual intercourse as a means of COVID-19 transmission → **I would rephrase this sentence to improve its understanding**
maybe something like → Other studies from Ethiopia and elsewhere showed that 12.38% and 7.7% of participants responded blood transfusion was a mean of COVID-19 transmission while 9% and 7.4% of participants answered sexual intercourse was another mean. [I’m sure **you can even make a better sentence**]

For sure, take away the parenthesis and don’t say “as a means” , “a “ is singular while “means” is plural.

372-373 and after →and after it
the government has deployed →has or had?

409
a) it needs → he/she needs
b) to believe that personally susceptible → to believe he/she is personally
c) susceptible to a health → susceptible to such health ??

415 Implies the more → Which implies?

429 COVID-19, in turn it helps → COVID-19, to probably help the

432 the pieces of evidence →the evidence revealed

433 in a home, in turn, would help → in a home, which would help

484 strategies by considering → strategies considering

488 in the study setting → in any setting.

FIG 1 It’s repeated, apostrophe incorrect “participants`". Has the year on it: 2021. I’m not used to see it on a table nor a figure.

Bias

- only people who attended the follow up clinic sesgo de gente preocupada por su salud
