# Supplementary material for: Compliance with COVID-19 preventive measures among chronic disease patients in Wolaita and Dawuro zones, Southern Ethiopia: A proportional odds model
Source: PLoS One. 2022 Oct 26;17(10):e0276553. doi: 10.1371/journal.pone.0276553 (PMC9604994; doi:10.1371/journal.pone.0276553)
Supplement: Attachment — Submitted filename: Plos_Ethiopia.docx [file pone.0276553.s003.docx]

I would like to acknowledge the effort of the authors to do this research. I agree this is an important topic to address especially as COVID-19 vaccination is not so high in Ethiopia and some African countries, we need to help patients to understand how to prevent COVID-19. Said that, I found confusing how they built the test and how they measured the different sections of the test. I think they have to make the information easier to read.

Title: I would recommend the short title adding the location.

Abstract: I would appreciate that they quote at least 1-2 preventive measures in the background to be able to fully understand their results.

Manuscript:

Lines 63-64, As the results are related with the socioeconomic condition of the patients, it would be interesting to add a sentence to describe as low socioeconomic status makes people more vulnerable to COVID-19.

Lines 88-90. It would be easier to understand the results if they described which category was more related to the preventive practices as they described the general characteristics (example: not sex but males, etc.).

Lines 119-124. This looks like a strategy for research or for practice in the future. I would add this sentence to the discussion.

I miss that the transmission of COVID-19 is described in the introduction, especially, because the airborne transmission has not been described in this article although many scientists agree about the airborne transmission and not only the droplets. <https://pubmed.ncbi.nlm.nih.gov/33865497/>

Some explanation about why the airborne transmission was not included in the text must be shared in the discussion.

Lines 128-134, please add the country. It would help to know the population that is assigned to the WSUCSH and DTGH, especially because after the sample size is calculated. If the facilities are public ones, it would be nice to say it here and not later in the text (137)

Also, as some patients have been chronic patients who were looked after more than 5 years, it would be nice to add it here.

Lines 136-141. I would simplify the text, a suggestion: “Inclusion criteria: patients who were > 18 years, they had chronic diseases (hypertension, diabetic mellitus, and bronchial asthma) and they were on follow-up in the hospitals during the data collection period. Whereas unable to communicate via any channel, and admitted patients were excluded”.

Lines 148-150 This information will be better located in the setting. On the other hand, are these clinics the only ones in the whole country, in the area? Please, clarify.

Lines 150-152, it would be clearer if they describe the flow of patients in the clinics in the setting section in line 128-134.

Lines 161-206. I found difficult to follow this section. I don’t know if I am wrong but I have understood that the authors created the test with sections of different surveys that have been administered in other studies. If this is the case, it would be nice to start with this clear information. I would suggest to add a table with the scores of each scale. It is difficult to interpret the results without a clear idea of how each variable was measured (169-206). Also finishing with the score of this final test.

Line 209. I would suggest to describe this section after sampling technique. I was confused reading line 162 about the pre-tested.

Line 292-296, As a suggestion: It would be interesting to know if patients who had several conditions are more prone to compliance the preventive measures.

The titles of the tables must be simplified. There is no need to describe the patients and the setting as both characteristics have been described in methods before.

In table 6, the average monthly income is difficult to understand for an international audience, please change it to USA dollars.

Lines 350-351 has already been said before.

Lines 357-362, it would be needed to compare with similar countries to make the text more interesting for an international audience.

Lines 370-375. I would like a little bit of context about the COVID-19 health promotion in Ethiopia. Was there any national campaign to explain the protection measures? What kind of education both hospitals offered to their patients? May be patients were not protecting themselves because anyone explained them clearly how to do it. As mass media or religious leaders seem to have a role in the community, it would be nice to quote if any of the leaders in the community or in the country took a proactive role to share preventive measures.

Lines 424-434. I think this is a key result, clean water is a basic need and this study gives another motive to support the access to clean water in each home.

Line 440-451. If we are going to discuss the impact of having internet access. There should be a reference in the introduction regarding the use of internet in Ethiopia. Secondly, it would be helpful to understand how people in the community are using it, do they use more social media (Facebook, WhatsApp, Telegram, etc.) than other sources of information?
