# Supplementary material for: Compliance with COVID-19 preventive measures among chronic disease patients in Wolaita and Dawuro zones, Southern Ethiopia: A proportional odds model
Source: PLoS One. 2022 Oct 26;17(10):e0276553. doi: 10.1371/journal.pone.0276553 (PMC9604994; doi:10.1371/journal.pone.0276553)
Supplement: Attachment — Submitted filename: Response to Reviewers.doc [file pone.0276553.s004.doc]

We would like to thank the editors and the reviewers for careful review of our manuscript and providing us with their comments and suggestions to further improve the quality of the manuscript. Accordingly, we have prepared the following responses to address the editors` and the reviewers` comments and suggestions.

- **Responses for** **editors` comments:**
- **Journal Requirements: “when submitting your revision, we need you to address these additional requirements.”**
- **Comment_1.** Please ensure that your manuscript meets PLOS ONE's style requirements, including those for file naming. The PLOS ONE style templates can be found at 
  <https://journals.plos.org/plosone/s/file?id=wjVg/PLOSOne_formatting_sample_main_body.pdf> and <https://journals.plos.org/plosone/s/file?id=ba62/PLOSOne_formatting_sample_title_authors_affiliations.pdf>
- **Response_1.** We have formatted the manuscript in accordance with PLOS ONE's style requirements, including file naming **(Please, see the uploaded Revised Manuscript with Track Changes and the Manuscript file).**
- **Comment_2.** Thank you for stating the following in the Acknowledgments Section of your manuscript:

"This study was funded by the Wolaita Sodo University. The funder had no role in the study design, data analysis, result interpretation, and decision to publish."

We note that you have provided funding information that is not currently declared in your Funding Statement. However, funding information should not appear in the Acknowledgments section or other areas of your manuscript. We will only publish funding information present in the Funding Statement section of the online submission form.

Please remove any funding-related text from the manuscript and let us know how you would like to update your Funding Statement. Currently, your Funding Statement reads as follows:

"This study was funded by Wolaita Sodo University with the wsu41/19/368 grant numbers. Received by TBG. The funders had no role in study design, data collection and analysis, decision to publish, or preparation of the manuscript."

Please include your amended statements within your cover letter; we will change the online submission form on your behalf.

- **Response_2.** We have removed any funding-related text from the manuscript (**Please, see the acknowledgements and funding sections of Revised Manuscript with Track Changes file**). So, please update our Funding Statement as follows: *This study was funded by the Wolaita Sodo University with the wsu41/19/368 grant numbers. Received by TBG. The funders had no role in study design, data collection and analysis, decision to publish, or preparation of the manuscript*.
- **Comment_3.** We note that you have indicated that data from this study are available upon request. PLOS only allows data to be available upon request if there are legal or ethical restrictions on sharing data publicly. For more information on unacceptable data access restrictions, please see [http://journals.plos.org/plosone/s/data-availability#loc-unacceptable-data-access-restrictions](http://journals.plos.org/plosone/s/data-availability#loc-unacceptable-data-access-restrictions).

In your revised cover letter, please address the following prompts:

a) If there are ethical or legal restrictions on sharing a de-identified data set, please explain them in detail (e.g., data contain potentially sensitive information, data are owned by a third-party organization, etc.) and who has imposed them (e.g., an ethics committee). Please also provide contact information for a data access committee, ethics committee, or other institutional body to which data requests may be sent.

b) **If there are no restrictions,** please upload the minimal anonymized data set necessary to replicate your study findings as either Supporting Information files or to a stable, public repository and provide us with the relevant URLs, DOIs, or accession numbers. For a list of acceptable repositories, please see [http://journals.plos.org/plosone/s/data-availability#loc-recommended-repositories](http://journals.plos.org/plosone/s/data-availability#loc-recommended-repositories).

We will update your Data Availability statement on your behalf to reflect the information you provide.

- **Response_3.** There are no restrictions on sharing our study`s data. Therefore, currently we have uploaded the minimal dataset necessary to replicate the study findings as Supporting Information files. Besides, we kindly request you to update the Data Availability statement on our behalf.
- **Responses for reviewers' comments:**
- **“Reviewer's Responses to Questions”**
  1. Is the manuscript technically sound, and do the data support the conclusions?
  The manuscript must describe a technically sound piece of scientific research with data that supports the conclusions. Experiments must have been conducted rigorously, with appropriate controls, replication, and sample sizes. The conclusions must be drawn appropriately based on the data presented.

Reviewer #1: Yes

Reviewer #2: Yes

**Response.** We appreciate that

2. Has the statistical analysis been performed appropriately and rigorously?

Reviewer #1: I Don't Know

Reviewer #2: Yes

**Response.** The statistical analyses were performed in accordance with PLOS ONE's SAMPL guidelines, the “Statistical Analyses and Methods in the Published Literature”. The required “General Principles for Reporting Statistical Methods” are followed. Accordingly;

- **Preliminary analyses:** Identifying any statistical procedures used to modify raw data before analysis such as collapsing continuous data into categorical data or combining categories or vice versa is done **(Please, see the instrument and measurements, and data management and analysis procedure sections)**.
- **Primary analyses**: Describing the purpose of the analysis, summarizing data using descriptive statistics, naming the statistical package used, and reporting the level of significance is performed (**Please, see the data management and analysis procedure section**).
- **Supplementary analyses:** Describing methods used for any ancillary analyses, such as testing of assumptions underlying methods of analysis is performed **(Please, see the data management and analysis procedure section).**

3. Have the authors made all data underlying the findings in their manuscript fully available?

The [PLOS Data policy](http://www.plosone.org/static/policies.action#sharing) requires authors to make all data underlying the findings described in their manuscript fully available without restriction, with rare exception (please refer to the Data Availability Statement in the manuscript PDF file). The data should be provided as part of the manuscript or its supporting information, or deposited to a public repository. For example, in addition to summary statistics, the data points behind means, medians and variance measures should be available. If there are restrictions on publicly sharing data—e.g. participant privacy or use of data from a third party—those must be specified.

Reviewer #1: Yes

Reviewer #2: Yes

**Response.** We appreciate that

4. Is the manuscript presented in an intelligible fashion and written in standard English?

PLOS ONE does not copyedit accepted manuscripts, so the language in submitted articles must be clear, correct, and unambiguous. Any typographical or grammatical errors should be corrected at revision, so please note any specific errors here.

Reviewer #1: Yes

Reviewer #2: Yes

**Response.** We appreciate that

- **“5. Review Comments to the Author”**
- **Comment.** Please use the space provided to explain your answers to the questions above. You may also include additional comments for the author, including concerns about dual publication, research ethics, or publication ethics. (Please upload your review as an attachment if it exceeds 20,000 characters)

Reviewer #1: Thought at the beginning I thought this paper wasn't interesting at all, I do believe it shows that knowing your population before taking any healthcare measure is a must. In Spain, when I ask my patients to wash frequently their hands I do not consider the possibility of not having access to a hand washing facility or if I give them written instructions to improve the preventive measure they can take to avoid covid I wouldn't think that 10% are unable to read. That's why I believe the authors conclusions are right but could be generalized to include something like "know your population before implementing any preventive measure"

- **Response.** We thank you so much for the acknowledgement and the suggestion. Accordingly, the suggested statement is incorporated in **the conclusions section of the revised manuscript**.

Reviewer #2: I would like to acknowledge the effort of the authors to do this research. I agree this is an important topic to address especially as COVID-19 vaccination is not so high in Ethiopia and some African countries, we need to help patients to understand how to prevent COVID-19. Said that, I found confusing how they built the test and how they measured the different sections of the test. I think they have to make the information easier to read.

- **Response.** We appreciate your acknowledgement and comment. In the current **revised manuscript**, we have revised the entire **data management and analysis procedure section.** Besides, we have moved all issues related with the tests from **results section** to **data management and analysis procedure section**.
- **Comment. “**Title: I would recommend the short title adding the location.”
- **Response.** We accepted the comment and now, the title is modified accordingly to PLOS ONE's a full title requirements.
- **Comment.** “Abstract: I would appreciate that they quote at least 1-2 preventive measures in the background to be able to fully understand their results.”
- **Response.** Now, some preventive measures are included accordingly in the **abstract** **section;** **introduction subsection.**
- **Manuscript:**
- **Comment.** “Lines 63-64, As the results are related with the socioeconomic condition of the patients, it would be interesting to add a sentence to describe as low socioeconomic status makes people more vulnerable to COVID-19.”
- **Response.** A sentence that describes the association between low socio-economic status and COVID-19 is included in the **introduction** **section.**
- **Comment.** “Lines 88-90. It would be easier to understand the results if they described which category was more related to the preventive practices as they described the general characteristics (example: not sex but males, etc.).”
- **Response.** Now, the specific characteristics of the participants that associated with the preventive practices are described (**Please, see the introduction section**).
- **Comment.** “Lines 119-124. This looks like a strategy for research or for practice in the future. I would add this sentence to the discussion.”
- **Response.** We accepted the comment and the suggested modification are made (**Please, see the first paragraph of discussion section**).

- **Comment.** “I miss that the transmission of COVID-19 is described in the introduction, especially, because the airborne transmission has not been described in this article although many scientists agree about the airborne transmission and not only the droplets. <https://pubmed.ncbi.nlm.nih.gov/33865497/>
  Some explanation about why the airborne transmission was not included in the text must be shared in the discussion.”
- **Response.** Now, the evidences that related with mode of transmission of COVID-19 are included in the **second paragraph** **of the introduction section**.
- **Comment.** “Lines 128-134, please add the country. It would help to know the population that is assigned to the WSUCSH and DTGH, especially because after the sample size is calculated. If the facilities are public ones, it would be nice to say it here and not later in the text (137). Also, as some patients have been chronic patients who were looked after more than 5 years, it would be nice to add it here.”
- **Response.** Now, the suggested amendment is made accordingly (**Please, see the study design, setting and period section**).
- **Comment.** “Lines 136-141. I would simplify the text, a suggestion: “Inclusion criteria: patients who were > 18 years, they had chronic diseases (hypertension, diabetic mellitus, and bronchial asthma) and they were on follow-up in the hospitals during the data collection period. Whereas unable to communicate via any channel, and admitted patients were excluded”.”
- **Response.** We thank you so much for the suggestion and now, the amendment is made accordingly (**Please, see the participants section**).
- **Comment.** “Lines 148-150 This information will be better located in the setting. On the other hand, are these clinics the only ones in the whole country, in the area? Please, clarify.”
- **Response.** We accepted the comment and the suggested information is now located in the setting (**Please, see the study design, setting and period section**).
- **Comment. “**Lines 150-152, it would be clearer if they describe the flow of patients in the clinics in the setting section in line 128-134.”
- **Response.** Now, the information related with patients flow is describe in the setting (**Please, see the study design, setting and period section**).
- **Comment. “**Lines 161-206. I found difficult to follow this section. I don’t know if I am wrong but I have understood that the authors created the test with sections of different surveys that have been administered in other studies. If this is the case, it would be nice to start with this clear information. I would suggest to add a table with the scores of each scale. It is difficult to interpret the results without a clear idea of how each variable was measured (169-206). Also finishing with the score of this final test.”
- **Response.** We have modified the entire instrument and measurements section. In the meantime, we tried to address the raised issues **in the instrument and measurements section (Please, see the uploaded Revised Manuscript with Track Changes and the Manuscript file).**.
- **Comment. “**Line 209. I would suggest to describe this section after sampling technique. I was confused reading line 162 about the pre-tested.”
- **Response.** **The data collection methods and quality assurance section** is now located after sampling technique (**Please, see the revised version of the manuscript**).
- **Comment. “**Line 292-296, As a suggestion: It would be interesting to know if patients who had several conditions are more prone to compliance the preventive measures.”
- **Response.** We completely agree with your suggestion. However, their relationship has already been checked during the selection of candidate explanatory variables for final model which was not shown a statistically significant relationship. As a result, this variable was not included in the table (previously named table 5, currently modified as table 4) (**please, see both the previous and the revised manuscript**).
- **Comment. “**The titles of the tables must be simplified. There is no need to describe the patients and the setting as both characteristics have been described in methods before.”
- **Response.** Now, the titles of the tables are simplified as much as possible (**Please, see the revised version of the manuscript**).
- **Comment. “**In table 6, the average monthly income is difficult to understand for an international audience, please change it to USA dollars.”
- **Response.** We accepted the comment and the average monthly income ETB is change to US Dollar in **Table 1,4 and 5** (**see the revised version of the manuscript**).
- **Comment. “**Lines 350-351 has already been said before.”
- **Response.** Now, the stated objective is removed from **the discussions section**.
- **Comment. “**Lines 357-362, it would be needed to compare with similar countries to make the text more interesting for an international audience.”
- **Response.** We accepted the comment and we have compared with similar countries (**Please**, **see the discussions section of the revised manuscript**).
- **Comment. “**Lines 370-375. I would like a little bit of context about the COVID-19 health promotion in Ethiopia. Was there any national campaign to explain the protection measures? What kind of education both hospitals offered to their patients? May be patients were not protecting themselves because anyone explained them clearly how to do it. As mass media or religious leaders seem to have a role in the community, it would be nice to quote if any of the leaders in the community or in the country took a proactive role to share preventive measures.”
- **Response.** We accepted the comment and the suggested information is now included (**Please**, **see the introduction section and the discussions section of the revised manuscript**).
- **Comment. “**Lines 424-434. I think this is a key result, clean water is a basic need and this study gives another motive to support the access to clean water in each home.”
- **Response.** We thank you so much.
- **Comment.** Line 440-451. If we are going to discuss the impact of having internet access. There should be a reference in the introduction regarding the use of internet in Ethiopia. Secondly, it would be helpful to understand how people in the community are using it, do they use more social media (Facebook, WhatsApp, Telegram, etc.) than other sources of information?
- **Response.** We accepted the comment and the suggested information is now included in **the introduction section of the revised manuscript**.

**We thank you so much!**
